# Supplementary material for: Supplemented nutrition decreases helminth burden and increases drug efficacy in a natural host–helminth system
Source: Proc Biol Sci. 2021 Jan 20;288(1943):20202722. doi: 10.1098/rspb.2020.2722 (PMC7893286; doi:10.1098/rspb.2020.2722)
Supplement: Electronic Supplementary Material: Nutrition and helminths in wild wood mice [file rspb20202722supp1.pdf]

Supplemented nutrition decreases helminth burden and increases drug efficacy in a natural host-helminth system

Amy R. Sweeny, Melanie Clerc, Paulina A. Pontifes, Saudamini Venkatesan, Simon A. Babayan, Amy B. Pedersen

Electronic Supplementary Material

Proceedings of the Royal Society B doi: 10.1098/rspb.2020.2722

1. Methods

Here we provide additional information on methods used in the field and in the laboratory experiment, as well as rationale for approach to age and body condition analysis [1].

1.1 Field Experiment

1.1.1 Experimental details

In 2015, we live-trapped three grids (1 supplemented grid, 2 control grids, 49 trapping stations per grid with 2 traps/station, 10m between each trap, for a total area of 3600m^2), while in 2016, we trapped four grids (2 supplementation and 2 control grids; with each grid set up as a 6x5 array of 30 trapping stations with 2 traps/station, 10m between each trap, for a total area of 2000m^2). All grids in both years were spaced a minimum of 50m from each other to minimise mouse movement between grids, and grids were randomly assigned to nutrition regimes prior to the start of the experiment. Experimental grids were supplemented TransBreed^{TM} –a high-nutrient, standard veterinary feed which is formulated for optimum breeding performance in laboratory mice and offers whole-diet nutrition to the wild mice in this study (20% protein, 10% fat, 38% starch, high content of micronutrients (Table S1). Anthelmintic treatment regime consisted of a combination of Ivermectin and Pyrantel pamoate—broad-spectrum anthelmintics which target adult and larval stages (Ivermerctin) and adult stages (Pyrantel) of *H. polygyrus* in both laboratory and wild mice [7-9]. Previous work in wild *A. sylvaticus* found that the combination of Ivermectin and Pyrantel at 9.4mg/kg and 100 mg/kg, respectively, efficiently cleared *H. polygyrus* infection for 12-16 days [10].During trapping, each trap was set with cotton wool bedding, and was baited with seeds, carrot, mealworms, and TransBreed^{TM} pellets (on supplemented grids only), set in the early evening (16.00-18.00) and then checked early the following morning.

1.1.2 Host data and sampling

At each capture, weight in grams and length in mm was measured for each individual. Body fat scores were assessed on a scale of 1-5 (emaciated-obese) by palpating the sacroiliac bones (back and pubic

bones) as detailed in [2]. After field data collection, body condition index (BCI) was calculated by obtaining the residuals of an ordinary least squares (OLS) regression of mass against length [3] for inclusion as a fixed effect in models of infection and immunity. We did not calculate body condition index for the colony mice as those measures would be obscured by the fact that they are, on average, much heavier compared to their wild counterparts.

Sex and reproductive status were assigned by visual examination of the genitals as male wood mice have a greater urogenital distance than females. Males were classed into the following reproductive categories: abdominal (testes non-visible); descended, or scrotal. Females were classed into the following reproductive categories: non-perforate vagina; perforate vagina; pregnant or lactating. Animals which are scrotal, pregnant, or lactating are considered reproductive for binary reproductive status assignment. Age of mice (classed as: juvenile, subadult, adult) was determined by weight and coat colour based on juvenile moulting patterns. Generally, juveniles weigh 10g or less, subadults weigh between 10-15g, and adults are 15g or heavier. Juveniles have a distinctly different coat colour (grey, compared to brown colour of adults), while subadults have an intermediate colour coat (grey/brown). Adult mice comprised 87.5% of all captures in our dataset (juveniles = 2.4%, subadults = 10.1%). Where available (for sacrificed animals), eyes were collected and stored in 10% formalin for dissection of eye lenses for higher resolution estimates of animal age. Eye lens mass has been shown to strongly correlate with age in many species (rodents and others), and has successfully been used to distinguish age classes for both laboratory and wild mice [4,5]. Eyes collected from sacrificed animals were removed from their container and left at room temperature for 5-10 minutes to allow the formalin to evaporate. Eye lenses were then extracted and dried at 70°C overnight. They were then weighed to the nearest mg using a precision balance. The combined weight of both eye lenses (log-transformed) for each individual were used as a proxy for age. We calculated the relationship between age and eye lens weight using wood mice of known ages from our colony to be (Figure S2):

$$Age\ (weeks) = \frac{eye\ lens\ weight\ (mg) - 0.043 * mouse\ weight\ (g) - 5.34}{0.152}$$

Blood samples were collected via mandibular bleed (first capture), tail snip (subsequent captures), or terminal bleed (sacrificed animals) a maximum of once per week, centrifuged at 12,000 rpm for 10 minutes for collection of the sera and then stored at -80°C upon returning to the laboratory. Faecal samples were collected from previously sterilised traps and then preserved in 10% formalin at 4°C until processing and 2-3 pellets from each faecal sample were stored at -80°C for faecal IgA antibody measures. Small intestine, caecum, and colon of each sacrificed individual were stored in 1X phosphate-buffered saline and dissected on the same day for counts of adult *H. polygyrus* worms present.

1.2 Laboratory experiment

1.2.1 Experimental details

We maintain a formerly-wild, but now lab-reared wood mouse colony in standard laboratory conditions at the University of Edinburgh. The colony has been in captivity for many generations, but the wood mice are purposely outbred to maintain genetic diversity. In this experiment, all mice were housed individually in ventilated cages (Techniplast, 1285L) with food and water ad libitum. *H. polygyrus* L3 larvae were isolated from the same Callendar Wood wild wood mouse population and were screened using PCR diagnostics to ensure the isolate was not contaminated with any other known mouse parasites or pathogens (IDEXX Bioresearch, Germany), and then passaged several times through colony-housed wood mice [6].

We carried out primary and secondary *H. polygyrus* inoculations in both control and supplemented diet groups. After diet acclimatisation, experimental mice were infected with 200 wild-derived *H. polygyrus* L3 in 150μL dH_2O via oral gavage (day 0). Fourteen days post-infection, half of the male and half of the female mice that had been challenged with *H. polygyrus* were randomly assigned to be treated and were given either anthelmintic drug treatment (same combination/doses as in field experiment) or a control dose of water. Control animals received equivalent volumes of water on day 0 and 14 (the primary infection and treatment timepoints). On day 21, all experimental mice were re-infected with 200 *H. polygyrus* L3 in 150μL H_2O via oral gavage to act as a secondary challenge, and all control mice were given a primary challenge with 200 *H. polygyrus* L3. On day 35 (14 days post-secondary challenge) all mice were culled and adult *H. polygyrus* in the small intestine were counted. Although anthelmintic drugs were administered to half of the experimental group before secondary challenge, there was no difference in worm clearance (as indicated by EPG) between drug-treated and control mice (Figure S8B) and thus they were combined within diet groups for these analyses. Over the course of this experiment, 5 mice exhibited weight loss over the threshold for our experimental protocol (not related to the diet supplementation or *H. polygyrus* infection) and were culled and removed from further analysis.

1.2.2 Diet Information

Wood mice in the laboratory colony were fed two different formulations of standard laboratory rodent chow. Control mice were fed with standard maintenance chow (RM1^{TM}), whereas supplemented mice were fed a specially-formulated chow (TransBreed^{TM}), which has been designed to include higher fat, protein, and micronutrient contents (Table S1). We selected those diets to approximate control and supplemented nutrition in our field experiment as closely as possible. Both diets were fed ad libitum and provided adequate maintenance nutrition. Compared to field animals, lab wood mice had higher body mass (Lab mean weight = 23.88g; Wild mean weight = 20.32g; T-test, $t = -2.99$ $p = 0.005$) and

better body condition than wild wood mice (Lab mean total fat score = 9.08/10; Wild mean total fat score = 5.7/10, Wilcoxon Rank-Sum test, $W = 127$, $p < 0.001$).

Table S1. Nutrition content of SDS chow used in laboratory experiment comparing *H. polygyrus* infection for mice on both a standard (RM1^{TM}) and supplemented (Transbreed^{TM}) diet. Micronutrients included are selected elements and vitamins which have previously been implicated in host response to helminth infection, however full calculated analysis of RM1^{TM} and Transbreed^{TM} nutritional content can be found at http://www.sdsdiets.com/pdfs/RM1P-E-FG.pdf & http://www.sdsdiets.com/pdfs/TransBreed.pdf.

Diet	Macronutrients (% by weight)			Micronutrients (quanity/ kg)				
	Protein	Fat	Starch	Zn	Fe	Se	Vit A	Vit E
RM1	14.38	2.7	44.97	35.75 mg	159.30 mg	298.99 µg	8554.27 iu	84.10 iu
Transbreed	20.07	10.05	38.48	80.42 mg	216.0 mg	429.81 µg	18368.9 iu	267.4 iu

1.2.3 Parasite inoculation

Third stage larvae (L3) of *H. polygyrus* were originally derived from wild wood mice from Callendar Park that were infected with *H. polygyrus* [6]. Since then they have been passaged approximately ten times through colony-housed wood mice at the University of Edinburgh. In order to extract *H. polygyrus* eggs from faecal samples, the pellets were broken up and mixed with inactivated charcoal to mimic soil. The charcoal-soil mix was spread thinly on moist filter paper maintained in petri dishes at 17C. Larvae started to hatch after approximately 12 days and were collected into sterilised water and kept at 4C until use. Prior to infections, larvae concentrations were adjusted to a final concentration of 200 L3/ 150µL. Infective doses were administered to mice via oral gavage.

1.2.4 Faecal Sampling

Faecal samples in the colony were collected by changing the cage bedding ~12 hr prior to each collection and collecting faecal pellets from the freshly-used bedding and then preserving the samples in 10% buffered formalin. A small sample of 2-3 pellets was also collected to measure faecal IgA.

1.3 Laboratory assays

1.3.1 Parasite counts

Saturated salt solution was added to formalin-preserved faecal samples to concentrate eggs on a coverslip, counted at 10X magnification, and adjusted by sample weight to the number of eggs per gram faeces (EPG). Adult *H. polygyrus* were counted from PBS-preserved small intestine sections within 5 hours of dissection.

1.3.2 Antibody assays

ELISAs were performed to measure (1) total faecal IgA concentration and (2) serum *H. polygyrus*-specific IgG1 titres for each mouse at each capture/sampling point. For IgA ELISA, a 3:1 volume of protease inhibitor solution (Complete Mini Protease Inhibitor Tablets, Roche) was added each faecal

sample and then homogenised. These faecal extractions were then incubated for 1hr at room temperature and then centrifuged at 12,000 rpm for 5min. Supernatants were separated from faecal pellets and stored at -80C. 96-well microplates (Nunc™ MicroWell™, ThermoScientifc™) were coated with goat anti-mouse IgA (Southern Biotech 1040-01, 2µg /ml) diluted in carbonate buffer overnight at 4C. Capture antibody was then flicked off plates and 4% BSA-TBS was added and incubated for 2hr at 37C to block non-specific binding sites. Faecal extracts were diluted 1:50 in 1% BSA TBS in triplicate and added to plates and incubated overnight at 4C. Two serial dilutions of IgA standard (BD Bioscience 3039828) were included on each plate as positive controls and to obtain curves for calculation of IgA concentration. Following incubation, plates were washed 3 times with TBS-Tween and 50µL of goat anti-mouse IgA-HRP (Southern Biotech 1040-05) diluted 1:4000 in 1% BSA TBS was added to each well. Plates were incubated for 1hr and washed 4x with TBS-Tween and 2x with ddH20. 50µL TMB substrate was added to each well and plates were developed for 7 minutes protected from light. After that, 50uL of 0.18M sulphuric acid was added to each well to stop the enzymatic reaction. Plates were read at 450nm and sample concentrations were determined by fitting a 4-parameter logistic regression to standard curves.

To determine *H. polygyrus*-specific IgG1 titres from blood sera, 96-well microplates were coated with *H. polygyrus* excretory-secretory antigen (HES, 1.0ug/ml; obtained from Amy Buck, University of Edinburgh, and Rick Maizels, University of Glasgow) diluted in carbonate buffer overnight at 4C. Capture antigen was then flicked off plates and 4% BSA-TBS was added and incubated for 2hr at 37C to block non-specific binding sites. Sera samples were prepared as twofold serial dilutions with a starting concentration of 1:100in 1% BSA TBS and added to plates, and incubated overnight at 4C. Two serial dilutions consisting of sera from *Mus musculus domesticus* experimentally infected with *H. polygyrus* in the laboratory were included on each plate as positive controls. Following incubation, plates were washed 3x with TBS-Tween and 50µL of goat anti-mouse IgG1-HRP (Southern Biotech 1070-05) diluted 1:2000 in 1% BSA TBS was added to each well. Plates were incubated for 1hr and washed 4x with TBS-Tween and 2x with ddH20. 50ul TMB substrate was added to each well and plates were developed for 7min protected from light, at which point 50µL of 0.18M sulphuric acid was added to each well to stop the enzymatic reaction. Plates were prepared with serial dilutions of reference and experimental samples, and a dilution factor of 1:200 was selected for calculation of relative antibody concentrations. Standardised IgG1 concentrations were calculated by plate as follows:

$$IgG1\ Standardised\ Concentration = \frac{Sample\ OD - Mean\ Plate\ Blanks}{Plate\ Positive\ Control\ OD - Mean\ Plate\ Blanks}$$

We assigned a value of 0 to samples for which the OD did not exceed 3x SD of control blanks as we considered them indistinguishable from no IgG1 response. We refer to both IgA and IgG1 values as ‘antibody concentration’.

Table S2. Model formulae for analyses of wild and laboratory experiments. EPG: eggs per gram faeces; dpi: days post-infection.

MODEL GROUP	SYSTEM	RESPONSE	EXPERIMENT TIMEPOINT	MODEL CLASS	MODEL FAMILY	FIXED EFFECTS*	INTERACTIONS	RANDOM EFFECTS
***H. POLYGYRUS* INFECTION**	Wild	EPG	First capture	GLMM	Negative Binomial	Body Mass + Reproductive status + Sex + Diet		Grid*Year
		EPG (mean)	Trapping duration			as above + Treatment	Diet:Treatment	Grid*Year
		Adult worm	End point			as above + Age	Diet:Treatment	Grid*Year
	Lab	EPG (peak)	Primary challenge	GLM	Negative Binomial	Body Mass + Sex + Age + Group + Diet		
		EPG (total)	Primary challenge			as above		
		Adult worm	End point			as above	Diet:timepoint	
**ANTIBODY RESPONSE**	Wild	IgA (total)	All captures	GLMM	Gaussian	Body condition index + Reproductive status + Sex, *H. polygyrus* infection + Diet + Year	Diet:Year	ID + Grid*Year
		IgG1 (specific)				as above		Grid*Year
	Lab	IgA (total)	14 & 21 dpi	GLMM	Gaussian	Sex + Age + *H. polygyrus* infection + Experiment Timepoint + Diet	Diet:timepoint	ID
		IgG1 (specific)	21 dpi	GLM		Sex + Age + *H. polygyrus* infection (n worms) + Diet		
**BODY CONDITION**	Wild	Body mass (g)	All captures	GLMM	Gaussian	Body length (mm) + Reproductive status + Sex + Year + Day + Diet + Treatment	Diet:Day	ID + Grid*Year
		Total fat score		CLMM		as above	Diet:Day	ID + Grid*Year
	Lab	Body mass (g)	All captures	GLMM	Gaussian	Sex + Age + Day + Diet	Diet:Day	ID
		Total fat score		CLMM		as above	Diet:Day	ID

Table S3. Description of fixed effects included in models

Term	System	Class	Description
Diet	Wild	Factor	Control (grids not supplemented); Supplemented (High quality pellets added to grids)
	Laboratory		Control (standard mouse chow); Supplemented (high-quality chow identical to those used in the field experiment)
Treatment	Wild & Laboratory	Factor	Control (water); Treated (combination anthelminthic)
H. polygyrus infection	Wild & Laboratory	Continuous	Endpoint: Number of adult worms; Other timepoints: Log, Eggs per gram (EPG)
Body Mass	Wild & Laboratory	Continuous	Mass (g)
Body Condition Index (BCI)	Wild	Continuous	Residuals of weight ~ length regression
Age	Wild	Continuous	Age proxy: Log, paired eye lenses mass
	Laboratory		Age in weeks
Reproductive status	Wild	Factor	Inactive (Males- Abdominal or Descended Testes; Females- nonperforate or perforate vagina); Active (Males- Scrotal; Females - Pregnant or Lactating);
Year	Wild	Factor	2015 Replicate; 2016 Replicate
Day	Wild & Laboratory	Continuous	Day of experiment
Timepoint	Laboratory	Factor	Day 14 or 21 post-infection
Group	Laboratory	Factor	Control (primary challenge only group); Experimental (primary and secondary challenge)
Grid	Wild	Factor	Spatial replicates: 2015 (Grids 1-3) or 2016 (Grids 1-4)

2. Raw Data Summary, Full Model Output & Model Variation Output

This section includes raw data summaries and full model output for further interpretation of data presented in the main text. Additionally, we describe the models using a 3-level factor for supplementation in the wild to more accurately classify mice who were captured on both supplemented and control grids. Only 18% (n = 16) of mice were captured on both grid types, but to test the possibility that effects of supplemented nutrition could be dependent on the time spent on these grids, we specified diet as ‘control’, ‘mix’, or ‘supplemented’, where ‘mix’ represented mice that were found on both control and supplement grids across the experiment. These models were very similar to main models, with the exception of a diet-by-treatment interaction as group sizes were not sufficient to fit this interaction. When compared to models with a 2-level diet group, this modification did not significantly improve the fit for models of EPG at first capture, post-treatment, or at end point (ΔAIC = 1.71, ΔAIC = -1.57, ΔAIC = 1.28, respectively; Fig S1) nor did the inclusion of this factor level change any of the main effects (Figures S1 and S4).

Table S4. Raw data *H. polygyrus* summary. Data represents mean eggs/gram (EPG) or number of worms (± SE), and sample sizes for the indicated group.

		Control (dH_2O)		Anthelminthic	
		Control	Supplemented	Control	Supplemented
Wild	First Capture (mean EPG)	100.34 (± 51.68); n=38	12.41 (± 3.20); n=52	na	na
	Trapping During (mean EPG)	43.68 (± 8.78); n=37	29.96 (± 11.07); n=46	29.02 (± 19.99); n=36	0.136 (± 0.136); n=48
	End point (Number of adult worms)	30.50 (± 7.99); n=6	12.21 (± 3.96); n=14	1.71 (± 0.89); n=7	0.13 (± 0.13); n=8

	Group	Primary Challenge		Secondary Challenge	
		Control	Supplemented	Control	Supplemented
Colony	Peak EPG	48.03(± 25.79); n=9	19.59 (± 8.42); n=10	1.76 (± 1.19); n=6	0.00 (± 0.00); n=7
	Sum EPG	63.86 (± 26.82); n=9	28.26 (± 11.38); n=10	2.72 (± 2.07); n=6	0.00 (± 0.00); n=7
	End point (Number of adult worms)	9.67 (± 3.38); n=3	12.33 (±4.91); n=3	28.33 (±6.50); n=6	6.71 (± 1.98); n=7

Table S5. Model estimates for fixed effects on wild *H. polygyrus* infection.

	**First Capture EPG**			**Trapping Duration EPG**			**End Point Number of adult worms**		
Term	Estimate (CI)	Std.error	p.value	Estimate (CI)	Std.error	p.value	Estimate (CI)	Std.error	p.value
Diet, Supplemented: Treated, Anthelminthic				-4.51 (-8.12 - -0.9)	1.84	**0.014**	-2.25 (-5.26 - 0.75)	1.53	0.142
Treated, Anthelminthic				-2.11 (-4.58 - 0.35)	1.26	0.092	-2.74 (-4.26 - -1.22)	0.78	**< 0.001**
Diet, Supplemented	-2.47 (-3.67 - -1.27)	0.61	**< 0.001**	-1.56 (-3.65 - 0.53)	1.07	0.145	-1.2 (-2.38 - -0.03)	0.6	**0.045**
Sex, Male	- 1.36 (-2.75 - 0.02)	0.71	0.054	0.25 (-1.59 - 2.09)	0.94	0.787	-1.16 (-2.58 - 0.26)	0.72	0.109
Reproductive, active	-1.63 (-2.91 - -0.36)	0.65	**0.012**	1.37 (-0.26 - 3.01)	0.83	0.100	-0.25 (-1.26 - 0.77)	0.52	0.632
Body Mass, g	0.19 (0 - 0.38)	0.10	**0.045**	0.3 (0 - 0.6)	0.15	0.053	0.21 (0.03 - 0.39)	0.09	**0.019**
Age (Eye lens weight, mg)							2.22 (0.41 - 4.04)	0.93	**0.016**
(Intercept)	2.51 (-0.33 - 5.36)	1.45	0.084	-2.46 (-7.84 - 2.91)	2.74	0.369	-6.32 (-11.48 - -1.16)	2.63	**0.016**
Marginal R^2	0.735			0.941			0.893		

Table S6. Model estimates for fixed effects on laboratory *H. polygyrus* infection

	**Primary Challenge Peak EPG**			**Primary Challenge Sum EPG**			**End Point (Primary & Secondary Challenge) Number of adult worms**		
Term	Estimate (CI)	Std.error	p.value	Estimate (CI)	Std.error	p.value	Estimate (CI)	Std.error	p.value
Diet, Supplemented: Secondary Challenge							-1.76 (-2.88 - -0.64)	0.57	**0.002**
Diet, Supplemented	-1.09 (-1.99 - -0.19)	0.46	**0.017**	-1.07 (-2.04 - -0.11)	0.49	**0.030**	0.21 (-0.73 - 1.14)	0.48	0.663
Group, Experimental	1.96 (0.86 - 3.05)	0.56	**<0.001**	2.22 (1.07 - 3.37)	0.59	**<0.001**	0.89 (0.07 - 1.71)	0.42	**0.033**
Sex, Male	-0.51 (-2.05 - 1.03)	0.79	0.515	0.06 (-1.62 - 1.74)	0.86	0.945	-0.08 (-1 - 0.83)	0.47	0.857
Body Mass, g	0.01 (-0.17 - 0.19)	0.09	0.902	-0.06 (-0.25 - 0.14)	0.10	0.570	0.06 (-0.04 - 0.16)	0.05	0.248
Age (Weeks)	-0.01 (-0.09 - 0.08)	0.04	0.875	-0.01 (-0.1 - 0.08)	0.05	0.810	-0.02 (-0.08 - 0.04)	0.03	0.428
(Intercept)	2.82 (-0.9 - 6.54)	1.9	0.138	3.52 (-0.46 - 7.5)	2.03	0.083	1.84 (-0.53 - 4.21)	1.21	0.127
Marginal R^2	0.647			0.671			0.398		

Table S7. Model estimates for fixed effects on body condition in wild and laboratory wood mice.

	Wild						Laboratory					
	Body Mass			Total Fat Score			Body Mass			Total Fat Score		
**Term**	**Estimate (CI)**	**Std. error**	**p.value**	**Estimate (CI)**	**Std. error**	**p.value**	**Estimate (CI)**	**Std. error**	**p.value**	**Estimate (CI)**	**Std. error**	**p.value**
Diet, Supplemented: Day	-0.16 (-0.28 - -0.03)	0.07	**0.018**	-0.15 (-0.25 - -0.04)	0.05	**0.005**	-0.01 (-0.05 - 0.03)	0.02	0.675	-0.08 (-0.29 - 0.13)	0.11	0.457
Diet, Supplemented	1.69 (0.48 - 2.91)	0.62	**0.006**	1.12 (0.38 - 1.86)	0.38	**0.003**	1.63 (-1.25 - 4.52)	1.47	0.267	5.02 (0.07 - 9.97)	2.53	**0.047**
Day	-0.02 (-0.12 - 0.08)	0.05	0.734	0.04 (-0.03 - 0.12)	0.04	0.281	-0.02 (-0.05 - 0.01)	0.01	0.161	0.02 (-0.12 - 0.16)	0.07	0.791
Year, 2016	1.13 (0.04 - 2.21)	0.55	**0.042**	-1.14 (-1.67 - -0.61)	0.27	**< 0.001**						
Treated, Anthelminthic	0.34 (-0.73 - 1.42)	0.55	0.532	-0.24 (-0.75 - 0.27)	0.26	0.363						
Sex, Male	0.67 (-0.5 - 1.83)	0.59	0.261	-0.38 (-0.93 - 0.17)	0.28	0.178	8.53 (5.67 - 11.39)	1.46	**< 0.001**	15.07 (8.07 - 22.08)	3.57	**< 0.001**
Reproductive, active	2.16 (1.26 - 3.05)	0.46	**< 0.001**	-0.12 (-0.7 - 0.46)	0.3	0.68						
Body length (scaled)	1.64 (1.08 - 2.2)	0.29	**< 0.001**									
Age (Weeks)							0.08 (-0.01 - 0.17)	0.04	0.078	-0.31 (-0.73 - 0.1)	0.21	0.139
Intercept	17.33 (15.76 - 18.9)	0.8	**< 0.001**				15.65 (12.43 - 18.87)	1.64	**< 0.001**			
Marginal R^2	0.388			0.162			0.732			0.776		
Conditional R^2	0.689			-			0.988			**0**.953		

Table S8. Model estimates for fixed effects on immune responses in wild and laboratory wood mice.

	**Wild**						**Laboratory**					
	**IgA, Total**			**IgG1, Specific**			**IgA, Total**			**IgG1, Specific**		
Term	Estimate (CI)	Std. error	p.value	Estimate (CI)	Std. error	p.value	Estimate (CI)	Std. error	p.value	Estimate (CI)	Std. error	p.value
Diet, Supplemented: Year, 2016 (wild)/ Time point (Lab)	6.31 (2.77 - 9.85)	1.81	**<0.001**	0.13 (-0.17 - 0.44)	0.16	0.390	-2.39 (-5.68 - 0.9)	1.68	0.155			
Diet, Supplemented	-0.31 (-2.69 - 2.07)	1.21	0.799	-0.1 (-0.27 - 0.08)	0.09	0.282	2.40 (0.42 - 4.38)	1.01	0.018	0.20 (0.11 - 0.3)	0.05	**<0.001**
Year, 2016 (wild) Timepoint, d21 (Lab)	-5.14 (-7.9 - -2.39)	1.41	**<0.001**	-0.05 (-0.29 - 0.2)	0.13	0.703	1.53 (-1.4 - 4.46)	1.49	0.306			
Body condition index	0.43 (0.17 - 0.69)	0.13	**0.001**	0.02 (0 - 0.03)	0.01	**0.022**						
Log, *H.polygyrus EPG*	0.36 (-0.11 - 0.82)	0.24	0.136	0 (-0.02 - 0.02)	0.01	0.913	-0.2 (-0.82 - 0.43)	0.32	0.538	0.00 (0.00 – 0.00)	0.00	0.442
Treated, Anthelminthic	1.91 (0.1 - 3.72)	0.93	**0.039**	0.05 (-0.09 - 0.2)	0.07	0.453				0.16 (-0.81 - 1.13)	0.50	0.746
Sex, Male	0.06 (-1.62 - 1.75)	0.86	0.941	0.04 (-0.1 - 0.18)	0.07	0.602	0.42 (-1.72 - 2.57)	1.09	0.699	-0.23 (-0.31 - - 0.15)	0.04	**<0.001**
Reproductive, active	1.12 (-0.7 - 2.94)	0.93	0.227	-0.02 (-0.11 - 0.08)	0.05	0.734						
Age, weeks							-0.42 (-0.9 - 0.05)	0.24	0.082	0.00 (-0.02 - 0.02)	0.01	0.804
Intercept	18.82 (16.07 - 21.57)	1.4	**<0.001**	0.32 (0.13 - 0.51)	0.1	**<0.001**	31.63 (20.88 - 42.38)	5.48	<0.001	0.93 (0.39 - 1.47)	0.28	**0.001**
Marginal R^2	0.211			0.050			0.246			0.995		
Conditional R^2	0.287			0.772			-			**-**		

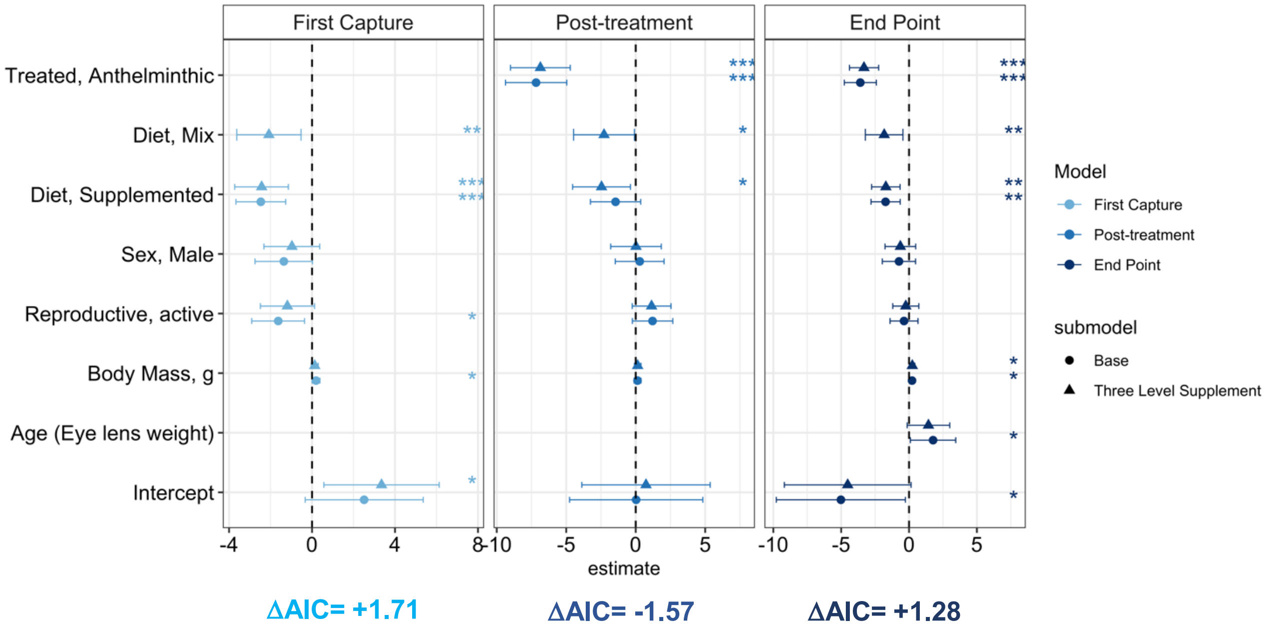

Figure S1. Effect size estimates from models investigating the effect of supplemented nutrition on *H. polygyrus* infection, accounting for individuals captured only a portion of time on supplemented grids (mix). Panels represent separate models for first capture, infection abundance (EPG); average EPG across two weeks for individuals captured beyond first capture and after assignment to treatment categories; end point burden (adult worm count) for individuals culled 12-16 days post first capture. Models represented are identical to those included in Fig 3 with the exception of the inclusion of an additional factor level in the supplemented nutrition explanatory variable. Points and ranges represent model estimates and 95% credibility estimates for each model. Asterisks indicate the significance of variables: ***, ** and * indicate P<0.001, P<0.01 and P<0.05 respectively. Change in AIC from main models is included in the bottom left of each panel.

3. Additional figures

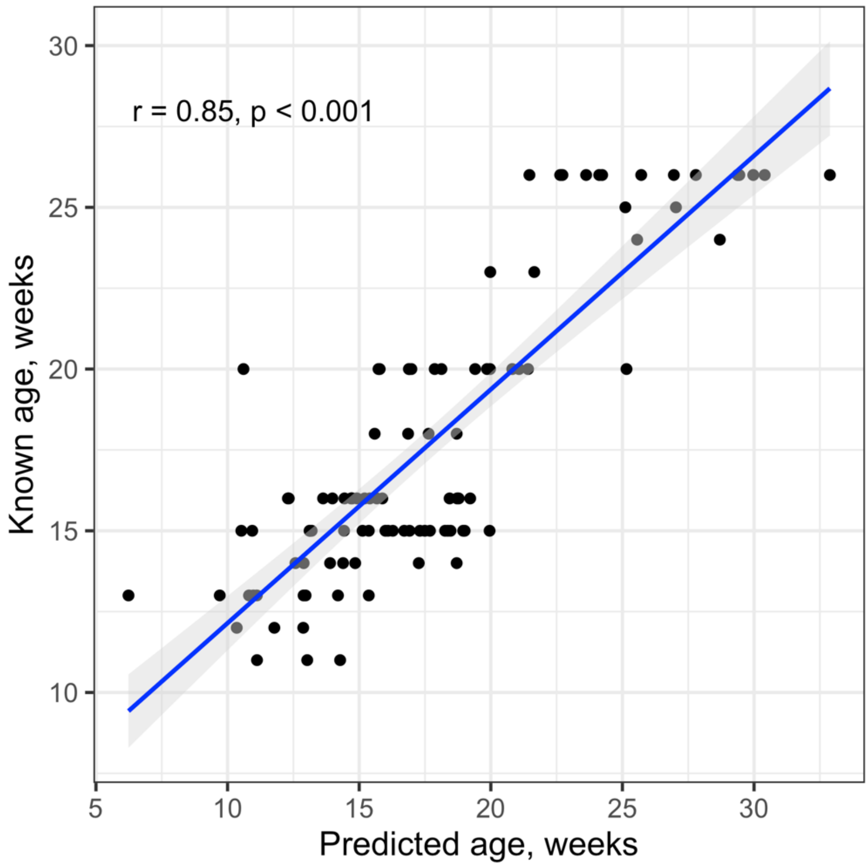

Figure S2. Correlation of wood mouse age predicted from eye lens weight and known age (in weeks) from colony wood mice. Pearson's r with 95% credibility intervals and significance of the correlation is included for both 2015 and 2016 data. Points have been jittered by 10% of raw values to aid in visualisation.

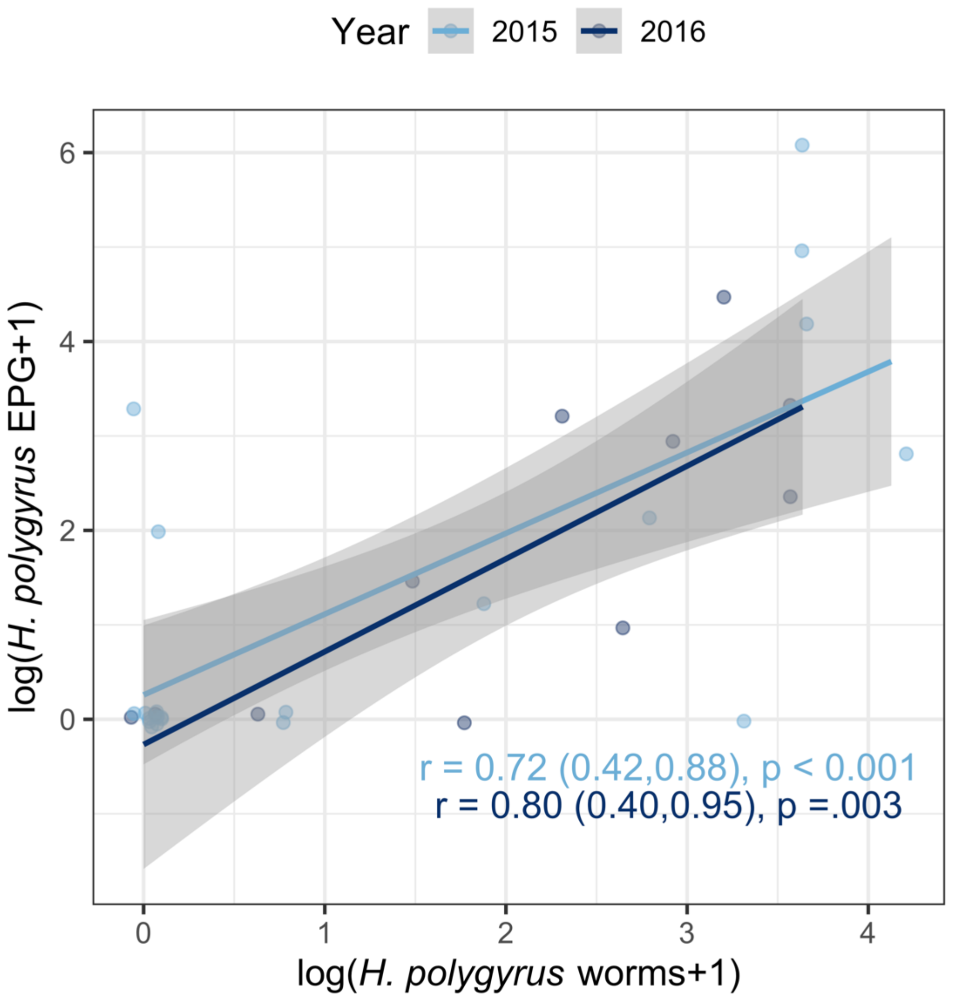

Figure S3. Correlation between *H. polygyrus* eggs/ gram faeces (EPG) and adult worm burden from culled animals where data was available for both metrics (N_i=36). Both values are log-transformed (EPG or count +1). Pearson's r with 95% credibility intervals and significance of the correlation is included for both 2015 and 2016 data. Points have been jittered by 10% of raw values to aid in visualisation.

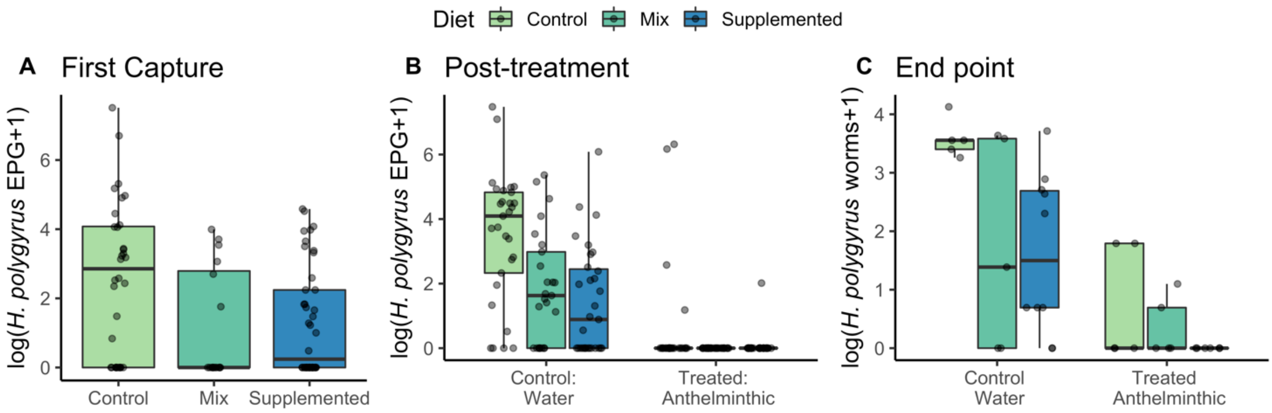

Figure S4. Effect of supplemented nutrition on H. polygyrus infection in wild wood mice, accounting for both individuals who were found exclusively on supplemented grids or individuals who were found on supplemented grids only a portion of the time ('Mix"). A. Infection intensity (EPG) at first capture, N=88 individuals. B. Mean EPG for all individuals captured beyond first capture and after assignment to treatment categories, N=62 individuals; 166 captures C. Adult worm burden at end point for culled individuals, N=36. Data represent log means and SEM for raw EPG data. Labels above bars indicate the number of observations for each group.

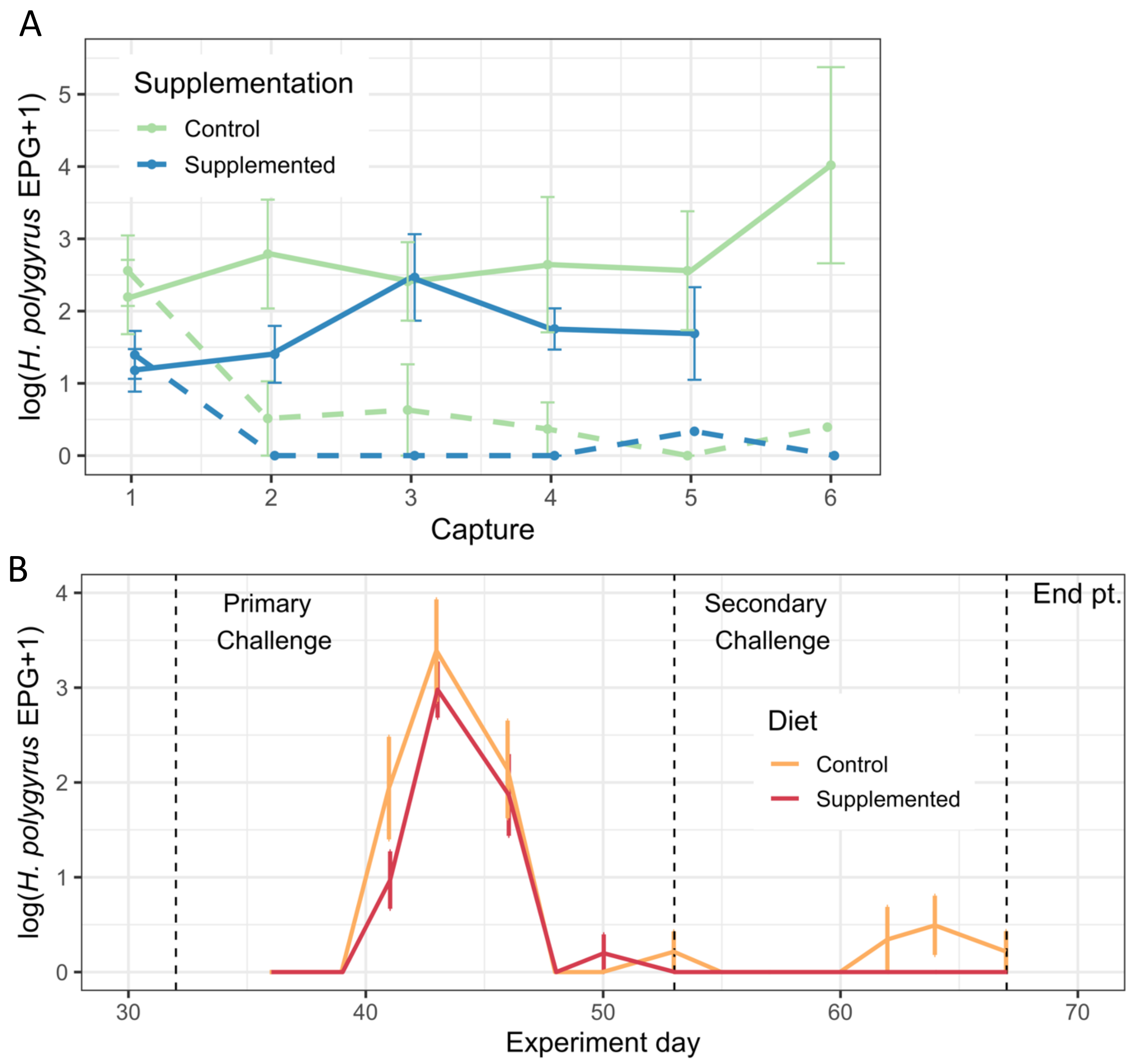

Figure S5. *H. polygyrus* intensity dynamics, as measured by eggs/gram over the course of (A) field experiment and (B) colony experiment. In both the wild and laboratory, supplemented mice maintain lower EPG for the majority of the experimental period. Data represent the log of EPG+1 and SE. Dashed lines in (A) represent individuals who were treated with anthelmintics. Dashed lines in (B) indicate infection and end timepoints of experiment as labelled.

Supplementary Information: References

1. Peig, J. & Green, A. J. 2010 The paradigm of body condition: a critical reappraisal of current methods based on mass and length. *Funct Ecology* **24**, 1323–1332. (doi:10.1111/j.1365-2435.2010.01751.x)

2. Ullman-Cullere, M. H. & Foltz, C. J. 1999 Body Condition Scoring: A Rapid and Accurate Method for Assessing Health Status in Mice. *Lab Anim Sci* **49**.

3. Peig, J. & Green, A. J. 2009 New perspectives for estimating body condition from mass/length data: the scaled mass index as an alternative method. *Oikos* **118**, 1883–1891. (doi:10.1111/j.1600-0706.2009.17643.x)

4. Rowe, F. P., Bradfield, A., Quy, R. J. & Swinney, T. 1985 Relationship Between Eye Lens Weight and Age in the Wild House Mouse (Mus musculus). *The Journal of Applied Ecology* **22**, 55. (doi:10.2307/2403326)

5. Augusteyn, R. C. 2014 Growth of the eye lens: I. Weight accumulation in multiple species. *Mol. Vis.* **20**, 410–426.

6. Clerc, M., Fenton, A., Babayan, S. A. & Pedersen, A. B. 2019 Parasitic nematodes simultaneously suppress and benefit from coccidian coinfection in their natural mouse host. *Parasitology* **6**, 1–11. (doi:10.1017/S0031182019000192)

7. Wahid, F. N., Behnke, J. M. & Conway, D. J. 1989 Factors affecting the efficacy of ivermectin against *Heligmosomoides polygyrus* (*Nematospiroides dubius*) in mice. *Vet Parasitol.* **32**, 325–340.

8. Ferrari, N., Cattadori, I. M., Nespereira, J., Rizzoli, A. & Hudson, P. J. 2004 The role of host sex in parasite dynamics: field experiments on the yellow-necked mouse *Apodemus flavicollis*. *Ecol. Lett.* **7**, 88–94. (doi:10.1046/j.1461-0248.2003.00552.x)

9. Ferrari, N., Cattadori, I. M., Rizzoli, A. & Hudson, P. J. 2009 *Heligmosomoides polygyrus* reduces infestation of *Ixodes ricinus* in free-living yellow-necked mice, *Apodemus flavicollis*. *Parasitology* **136**, 305–316. (doi:10.1017/S0031182008005404)

10. Clerc, M., Babayan, S. A., Fenton, A. & Pedersen, A. B. 2019 Age affects antibody levels and anthelmintic treatment efficacy in a wild rodent. *Int J Parasitol Parasites Wildl.* **8**, 240–247. (doi:10.1016/j.ijppaw.2019.03.004)
